# Social marketing and behavioral change

**DOI:** 10.22336/rjo.2021.21

**Published:** 2021

**Authors:** Dodu Gheorghe Petrescu, Laura Carina Tribus, Raluca Raducu, Victor Lorin Purcarea

**Affiliations:** * Department of Marketing and Medical Technology, “Carol Davila” University of Medicine and Pharmacy, Bucharest, Romania; ** Gastroenterology Department, Bucharest Emergency University Hospital, Bucharest, Romania

**Keywords:** attitudes, behavior, change, habits, social marketing

## Abstract

Social marketing is an arranged cycle for impacting change. With its marketing and exploring segments, publicizing and customer advancement (counting situating, division, imaginative system, message plan and testing, media technique and planning, and successful following), social advertising can assume a focal part in themes like wellbeing and climate. Social marketing consolidates the best components of conventional ways to deal with social change in an incorporated arranging and activity structure and it utilizes progresses in correspondence innovation and advertising abilities. Moreover, it uses marketing methods to create conversation and advance data, perspectives, qualities, and practices. Thus, it adds to establishing an environment helpful for social and conduct change.

With the separation of social frameworks, there have been endeavors to advise, persuade, impact, spur, acquire acknowledgment of new followers to specific arrangements of thoughts, elevate causes and win certain gatherings, reinforce certain practices, or transform them. In Ancient Greece and Rome, crusades were dispatched to free the slaves. In England, during the Industrial Revolution, crusades were coordinated to nullify account holders’ detainment facilities, award ladies’ democratic rights and dispense with kid work. Striking social change crusades in nineteenth century America included nullification, frugality, boycott, and suffragette developments, just as a buyer development for governments to direct the nature of food and medications. As of late, crusades have been dispatched in research fields like wellbeing advancement (against smoking, security, drug misuse, driving drunk, AIDS, nourishment, state of being, vaccination, bosom malignancy screening, emotional well-being, breastfeeding, family arranging), climate (more secure water, cleaner air, energy preservation, protection of public stops and woods), schooling (education, school standing), financial matters (boosting abilities and preparing, drawing in financial backers, reviving more established urban communities) and different issues like abusive behavior at home, common liberties and prejudice.

In this context, it was necessary for the marketing discipline to give a particular attention to social well-being and led to the emergence and development of social marketing, created by Kotler and Zaltman in 1971, an independent discipline, which aims to apply the commercial marketing toolkit to obtain social benefits [**1**,**2**]. By consulting behavioral sciences and sociology, the parents of marketing have given more definitions for social marketing. Social marketing is a program planning process that promotes the voluntary behavior of the target audience, delivering the benefits they want, reducing the obstacles it worries and using conviction to motivate their participation in the program’s work [**3**].

Social marketing is the utilization of business promoting innovations to the investigation, arranging, execution and assessment of projects intended to impact the willful practices of the intended interest group to improve their own and cultural prosperity [**4**]. 

Social marketing is the use of advertising standards and methods to impact the intended interest group to intentionally acknowledge, reject, adjust, or forsake conduct to help people, gatherings, or society all in all [**5**].

Social marketing is a set of evidence-based and experience-based concepts and principles that provide a systematic approach to understanding behavior and modifying it for the social good [**6**].

Over time, social marketing has gained global recognition and it is seen as the key to achieving social change by affecting personal behaviors [**7**]. Despite research and practices remarkably developed in the field, social marketing specialists have not yet reached the potential needed for effective behavioral change work [**8**,**9**]. The main reason is that, by emphasizing traditional marketing tools and information campaigns, they overestimated the power to target individual behaviors, which has a limited effect on real change [**10**]. This is mainly because external factors influence consumer choices and actions and, without altering them, no transition is possible [**11**].

Recent studies have shown that individuals and backgrounds constantly affect one another by shaping societal practices [**12**], and the key to changing human behavior lies in the dynamic system of these interactions [**10**].

The understanding of these interactions is especially important for working with sustainable lifestyles. Their adoption by people depends to a large extent on the infrastructure available [**12**]. On the other hand, sustainable lifestyles have challenging differences. First, they are generally seen not as normal in society, but rather as radical, and people tend to imitate the majority [**13**]. Secondly, the results of sustainable actions are intangible and on long-term, which make them difficult to be assessed [**14**]. Thus, sustainable lifestyles are incorporated into complex systems, in which both individual factors and physical and social factors play a defining role in their adoption. 

The behavior change is not an isolated process, but happens at the social level, involving interpersonal channels of communication [**15**,**16**]. At the same time, there is a diverse set of environmental barriers that constantly alter human choices [**17**]. However, social marketing campaigns tend to overlook these factors and are based on information campaigns that use traditional marketing principles and are intended to promote behavior in a similar way to products. Using this issue as an explanation for the failure of many behavior-changing initiatives, McKenzie-Mohr and Schultz (2014) suggested an alternative approach that has proven to be effective with a broad set of behaviors.

Behavior barriers can be internal (e.g., lack of knowledge/ skills) and external (e.g., lack of infrastructure) and can vary widely between individuals and situations. To overcome these barriers and encourage behavior change, strategies are developed based on extensive research into the social sciences. 

The most common competitors in social marketing are the consumer’s inertia and tendency to resist behavioral changes and continue with their current way of life. Another common and serious form of competition comes from commercial marketing that markets unhealthy and antisocial products, such as tobacco companies. Competition may also come from other social marketing professionals who market similar or alternative social marketing products [**18**]. 

Decades of research in social psychology, cognitive science, ecology, and cultural evolution have shown that human behavior is influenced by people’s environments, habits, abilities, and attitudes. In turn, these behaviors alter the environment and can be learned and socially transmitted.

The behavior change is very relevant to health because more than 50% of diseases are caused by human behavior [**19**]. The process of behavior change is much more complex than people originally thought. One of the key issues seems to be overestimating the significance of education and evidence in behavior change. People often do not change their behavior, even though they know they should and why they should do it. Behavioral change is an iterative process. 

Social marketing is also explored as a method of social innovation, a framework to increase the adoption of evidence-based practices among professionals and organizations, and as a basic skill for public sector managers and social entrepreneurs. It is seen as an approach to design more effective, fair, and sustainable approaches to enhance social well-being, which extends beyond individual behavior change, including the creation of positive changes in social networks and social norms, companies, markets, and public policies. 

**Conflict of Interest statement**

The authors state no conflict of interest.

**Acknowledgments**

None.

**Sources of Funding**

None.

**Disclosures**

None.
